# A dynamic meshing transmission dataset for manufacturing quality inspection of electric vehicle reducer gears

**DOI:** 10.1038/s41597-026-06885-1

**Published:** 2026-02-24

**Authors:** Dong Guo, Junjie Yang, Honglin Li, Yingjie Huang, Xiaoxiang Long, Yu Xin, Ming Li

**Affiliations:** 1 Key Laboratory of Advanced Manufacturing Technology for Automobile Parts, Ministry of Education, Chongqing University of Technology, Chongqing, 400054 China; 2 College of Vehicle Engineering, Chongqing University of Technology, Chongqing, 400054 China; 3 College of Mechanical Engineering, Chongqing University of Technology, Chongqing, 400054 China

**Keywords:** Mechanical engineering, Scientific data, Computer science

## Abstract

The NVH (Noise, Vibration, Harshness) performance of electric vehicle reducer gears directly affects the NVH level of the whole vehicle. However, the existing single gear quality detection methods based on tooth surface waviness are faced with two major challenges, which are unable to detect quickly and cannot fully characterize the real performance. This work introduces the first real industrial dataset for manufacturing quality inspection of electric vehicle reducer gears to solve these two challenges and provide data benchmarks. This dataset covers the dynamic meshing transmission data of five types of manufacturing offline gears (healthy gears, slight bump gears, leaky grinding gears, and two kinds of ghost order whine gears). And a variety of uncertain factors in the manufacturing process (different machine tools, different batches, different device control parameters, etc.) are introduced to avoid the influence of laboratory ideal conditions on signal characteristics. The data quality of this dataset was evaluated using a 1D-CNN classifier and benchmarked against WTGCM, MCC5-THU, and AGFD, where it exhibited superior performance.

## Background & Summary

The International Energy Agency (IEA) predicts that electric vehicle sales will exceed 20 million in 2025, accounting for a quarter of total vehicle sales^1^. It is foreseeable that as the global electric vehicle market continues to expand, the manufacturing quality requirements of its key components will also be significantly improved. Among them, the reducer is the core component of the power transmission. After the electric vehicle loses the noise masking effect of the engine, the vibration noise generated by the meshing of the gear system directly affects the NVH (Noise, Vibration, Harshness) performance of the vehicle. Therefore, it has become one of the key technical paths to enhance the competitiveness of the electric vehicle market to implement the manufacturing quality inspection of the offline gear from the source and control its NVH quality. However, there is no publicly available gear dataset from the production line to support the development of NVH performance testing technology and evaluation methods for gears.

Usually, the manufacturing enterprises of electric vehicle reducer gears adopt the method of batch sampling to carry out Fourier measurement of the tooth surface of the lower gear to evaluate the manufacturing quality of the gear. Tooth surface Fourier measurement can detect whether there is an abnormal waviness order on the gear tooth surface. However, due to the long detection period (The inspection time for a single gear is approximately 40 to 60 minutes), it is impossible to perform full batch inspection, resulting in unqualified gear assembly on the reducer, which will cause serious NVH problems and cause significant economic losses. In order to solve the above problems, this work evaluates the manufacturing quality of gears by obtaining the vibration data of dynamic meshing transmission of gear pairs. This method not only significantly shortens the detection time (It only takes about one minute to inspect a pair of gear pairs), supports batch full inspection, but also effectively avoids the problem that there is a significant difference between the evaluation results and the actual performance of the gear after assembly^2^. At the same time, based on this method, this work collects a large number of vibration data of the first-stage gear pair of the electric vehicle reducer during dynamic meshing transmission on the field of gear production, and forms a dynamic meshing transmission dataset (DMTD^3^) for manufacturing quality inspection of electric vehicle reducer gears.

For some currently available gear inspection datasets obtained under laboratory conditions, such as WTGCM^4^, MCC5-THU^5^, AGFD^6^, the typical failure gear state types they contain, such as missing teeth, cracks, spalling, and wear, do not conform to the gear manufacturing situation. The low-speed and low-torque test conditions under steady state do not conform to the actual application scenarios of electric vehicle reducer gears. The gearbox as a test object is affected by more coupling than a separate gear pair. Therefore, these datasets are not suitable for manufacturing quality inspection of electric vehicle reducer gears. Moreover, the gear vibration feature extraction model, intelligent diagnosis model and signal processing method proposed and verified based on such data are prone to the phenomenon of generalization ability attenuation in practical application. A summarized comparison (See Table 1) between DMTD and previous datasets is provided to demonstrate the advantages of DMTD.

**Table 1:** Comparisons with other datasets.

	WTGCM	MCC5-THU	AGFD	DMTD
Test object	Gearbox	Gearbox	Gearbox	Gear pair
Test condition	steady	Steady/variable	steady	variable
Speed range (r/min)	1,800	500 ~ 3,000	250 ~ 1,500	100 ~ 2,450
Load range (Nm)	—	5/10/15/20	0.12/0.71/1.31	−30/30
Sampling rate (kHz)	40	12.8	16	12.8
Abnormal source	Laboratory prefabrication	Laboratory prefabrication	Laboratory prefabrication	Manufacturing process
Anomaly visibility	Obvious	Obvious	Obvious	Not obvious/difficult to observe

It is hard to fairly compare different datasets, but we list some characteristics here as a rough reference.

The DMTD introduced in this work is specifically for gear manufacturing quality inspection and NVH performance evaluation. A variety of processing uncertainty factors are fully considered (different machine tools, different batches, different device control parameters, etc. The gear samples were sourced from various models of gear grinding machines from manufacturers such as NIDEC and CHMTI. The control parameters of each grinding machine are not exactly the same, but all the products meet the design requirements for gears. The collection of DMTD is a long-term process, thus including data of gears from different processing batches.), and the influence of idealized test conditions on signal characteristics in laboratory scenarios is avoided^7^. The DMTD encompasses five categories of gear data (healthy gears, slight bump gears, leaky grinding gears, and two kinds of ghost order whine gears). These data were collected under acceleration and deceleration conditions ranging from 100 to 2,450 r/min, ensuring a high level of completeness and engineering relevance. The DMTD serves as a foundational dataset with practical engineering implications for the robustness evaluation of algorithms used in gear manufacturing quality detection and for the validation of NVH performance prediction models. Additionally, it aids in assessing the model’s capability for high-order feature decoupling and weak signal perception.

**Advantages of the DMTD:**

- Dynamic characterization of microscopic defects on the tooth surface: Breaking the limitation that the existing datasets is only for prefabricated macro-faults, the gear data with abnormal tooth surface waviness caused by manufacturing was systematically collected for the first time. By constructing the direct mapping relationship of “machining anomaly - NVH response”, the data gap of NVH traceability analysis in gear manufacturing process is filled, which provides a new research dimension for precision machining process optimization, and also helps to train the fault diagnosis model to make it have better generalization ability and robustness.
- High-fidelity high-speed dynamic meshing characteristics: Compared with the multi-stage transmission gearbox datasets, the test object of DMTD is the first-stage gear pair of the electric vehicle reducer, which reduces the strong interference of the multi-stage transmission, thus improving the accuracy of the dynamic meshing transmission performance of the gear, and facilitating in-depth study of the high-speed dynamic meshing characteristics of the gear pair.
- Coverage of manufacturing scenarios: All data are measured at the first meshing transmission after the gear pair is manufactured, and a variety of uncertain influencing factors of processing (different machine tools, different batches, different device control parameters, etc.) are introduced to truly reflect the manufacturing quality of the gear. It avoids the limitations of idealized fault characterization under laboratory conditions and brings more challenges to data processing, analysis, and diagnosis.

**The deficiencies of the DMTD:**

The detection object of DMTD is the electric vehicle reducer gear actually produced by the enterprise according to the order. There are no typical failure types such as missing teeth, cracks, spalling and wear during the whole life cycle operation. Therefore, DMTD lacks detection data under typical failure modes of gears.

**The improvement of the DMTD:**

More abundant test conditions are helpful to the in-depth analysis of gear meshing transmission characteristics. In addition, compared with the vibration response, the transmission error signal is closer to the source, the interference of the transmission path is smaller, the influence of resonance is less, and the corresponding relationship with the simulation is better^8^. In the future, the transmission error test will be introduced to assist the research of gear manufacturing quality detection in the direction of multi-sensor data fusion.

## Methods

This section describes in detail the equipment used in the process of dynamic meshing transmission test of gear pair to obtain data, the experimental process and the state of the test gear.

### Test preparation

**Fig. 1:**
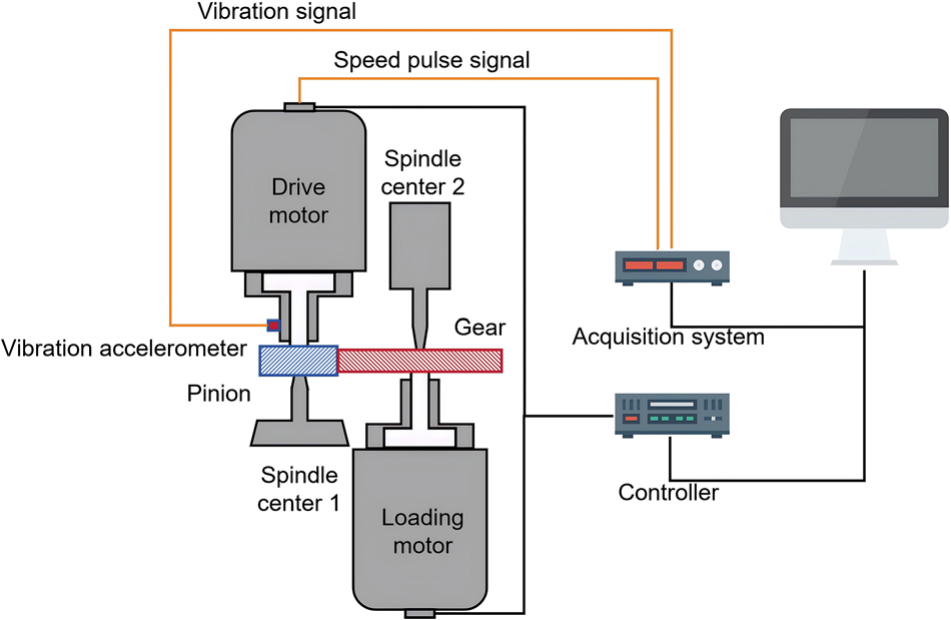
The working principle of the dynamic meshing transmission test equipment.

**Fig. 2:**
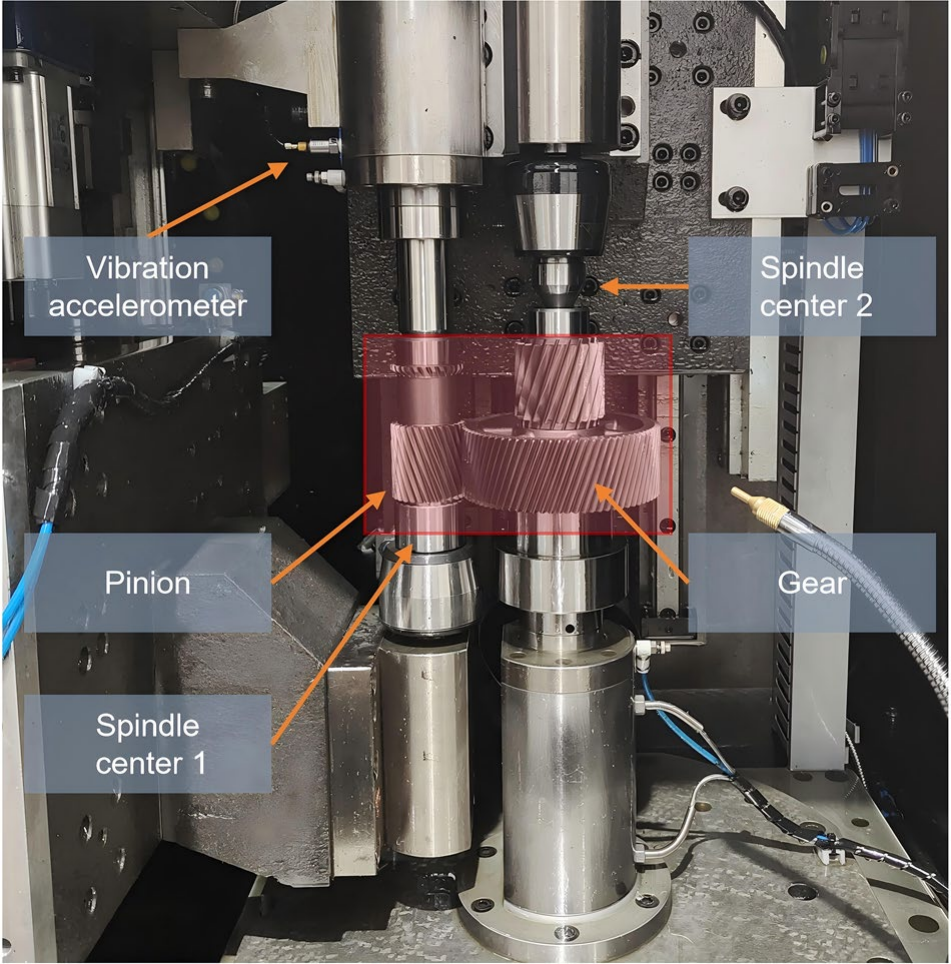
The dynamic meshing transmission test equipment.

The working principle of the dynamic meshing transmission test equipment is illustrated in Fig. 1, which includes a drive motor, a loading motor, a unidirectional vibration acceleration sensor, a controller, and a data acquisition system. The test equipment and the gear pair under test are presented in Fig. 2. Detailed specifications of the test equipment and the tested gear pair are summarized in Table 2.

**Table 2:** The specific parameters of the test equipment and the gear pair under test.

	parameter	value
1	rated power of the drive motor	10.9 kW
2	rated speed of the drive motor	2,300 r/min
3	rated torque of the drive motor	45 Nm
4	maximum speed of the drive motor	4,600 r/min
5	maximum torque of the drive motor	85 Nm
6	rated power of the loading motor	17 kW
7	rated speed of the loading motor	1,750 r/min
8	rated torque of the loading motor	93 Nm
9	maximum speed of the loading motor	4,600 r/min
10	maximum torque of the loading motor	145 Nm
11	test range of the vibration accelerometer	0 ~ 50 g
12	sensitivity of the vibration accelerometer	100 mV/g
13	frequency range of the vibration accelerometer	2 ~ 10 kHz
14	number of teeth on the pinion	27
15	number of teeth on the gear	79
16	Assembly center distance	87 mm
17	module	1.54
18	pressure angle	18 °
19	helix angle	20 °

The unidirectional vibration acceleration sensor is used to measure the vibration signal of the gear pair meshing transmission, and is placed horizontally at the main shaft of the driving motor. Because the characteristics of non-stationary signals are often related to speed changes, the speed signals are collected synchronously. The speed signals are obtained from the internal grating of the drive motor and recorded in the form of speed pulses.

### Experimental procedure

**Fig. 3:**
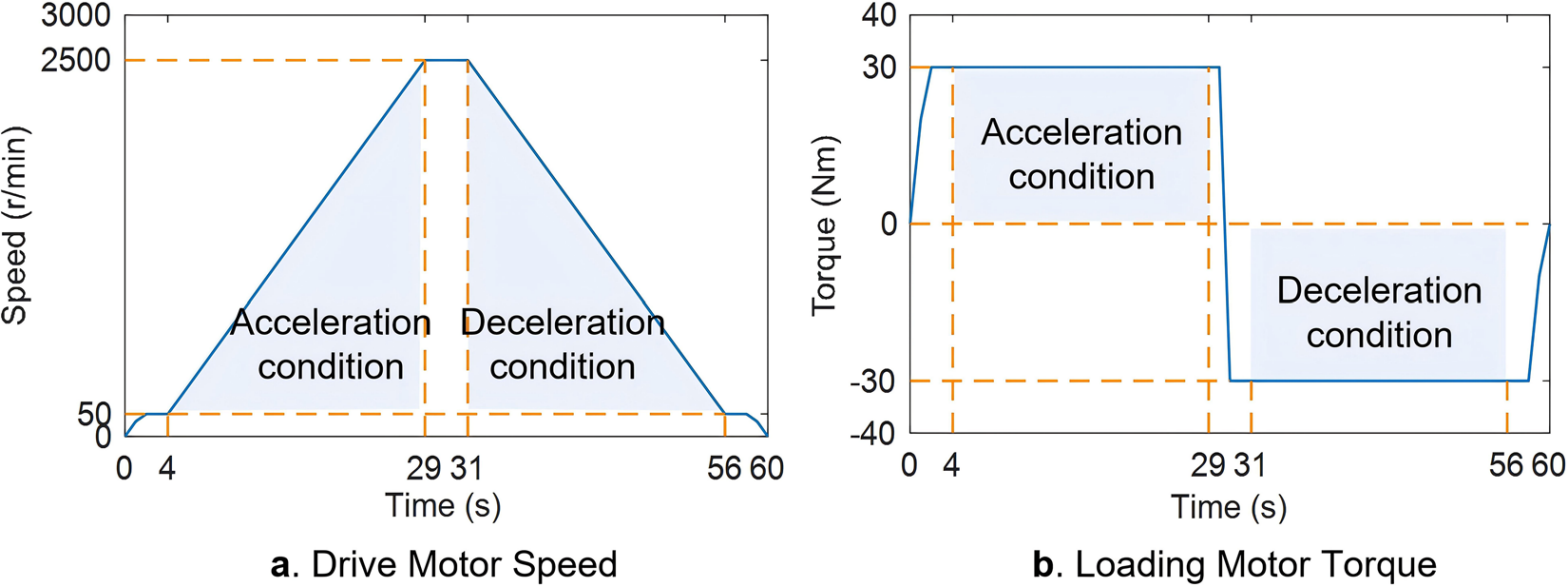
Test Conditions.

In order to stimulate a variety of NVH responses of the gear pair in the dynamic meshing transmission process, the sampling rate of the vibration acceleration sensor is set to 12.8 kHz after experimental verification, considering the data size and quality (Since the focus of DMTD is on ghost order whine gears, its characteristic frequency is usually between one times the gear meshing frequency (GMF) and five times the gear meshing frequency. Taking the 62nd ghost order whine gear as an example, when the pinion reaches the maximum testing speed (2,500 r/min), the shaft frequency is 41.67 Hz, and the fault frequency is 41.67 × 62 = 2.583 kHz. Additionally, Based on its number of teeth being 27, the 5 × GMF can be calculated to be 5.625 kHz. Therefore, according to the Nyquist theorem, a sampling rate of 12.8 kHz is sufficient to capture the relevant information of the abnormal gear.). The test conditions include acceleration and deceleration under different loads, and fully simulate the actual operating conditions of the gear. The time-varying curves of the test speed and torque are shown in Fig. 3.

The specific application process during the test is as follows:

Acceleration condition: The drive motor is accelerated to 50 r/min, and the loading motor provides a constant load of 30 Nm. After the load is stable, the drive motor is uniformly accelerated from 50 r/min to 2,500 r/min within 25 seconds. The detailed changes are as Eq. (1):

1 $${\rm{Acceleration}}\,{\rm{condition}}\left\{\begin{array}{c}{Speed}=50+98t\\ {Torque}=30\end{array}\right.0\le t\le 25$$

Considering the unstable interference at the beginning and end of acceleration, the vibration and speed pulse signals in the speed range of 100 r/min ~ 2,450 r/min are recorded.

Deceleration condition: After the drive motor accelerates to 2,500 r/min, the load motor switches the torque to −30 Nm. After the load is stable, the drive motor decreases from 2,500 r/min to 50 r/min in 25 seconds. The detailed changes are as Eq. (2):

2 $${\rm{Deceleration}}\,{\rm{condition}}\left\{\begin{array}{c}{Speed}=2500-98t\\ {Torque}=-30\end{array}\right.0\le t\le 25$$

Similarly, the vibration and speed pulse signals in the speed range of 2,450 r/min ~ 100 r/min are recorded.

In order to further verify the test results and form a category label, after the test is completed, the measured gear pair is sent to the Klingelnberg Gear Precision Measurement Center to complete the Fourier measurement of the tooth surface, and then sent to the Original Equipment Manufacturer (OEM) for assembly EOL detection (End Of Line). According to the measurement results and test results, the final category label of the measured gear is formed.

### Test gear condition

**Fig. 4:**
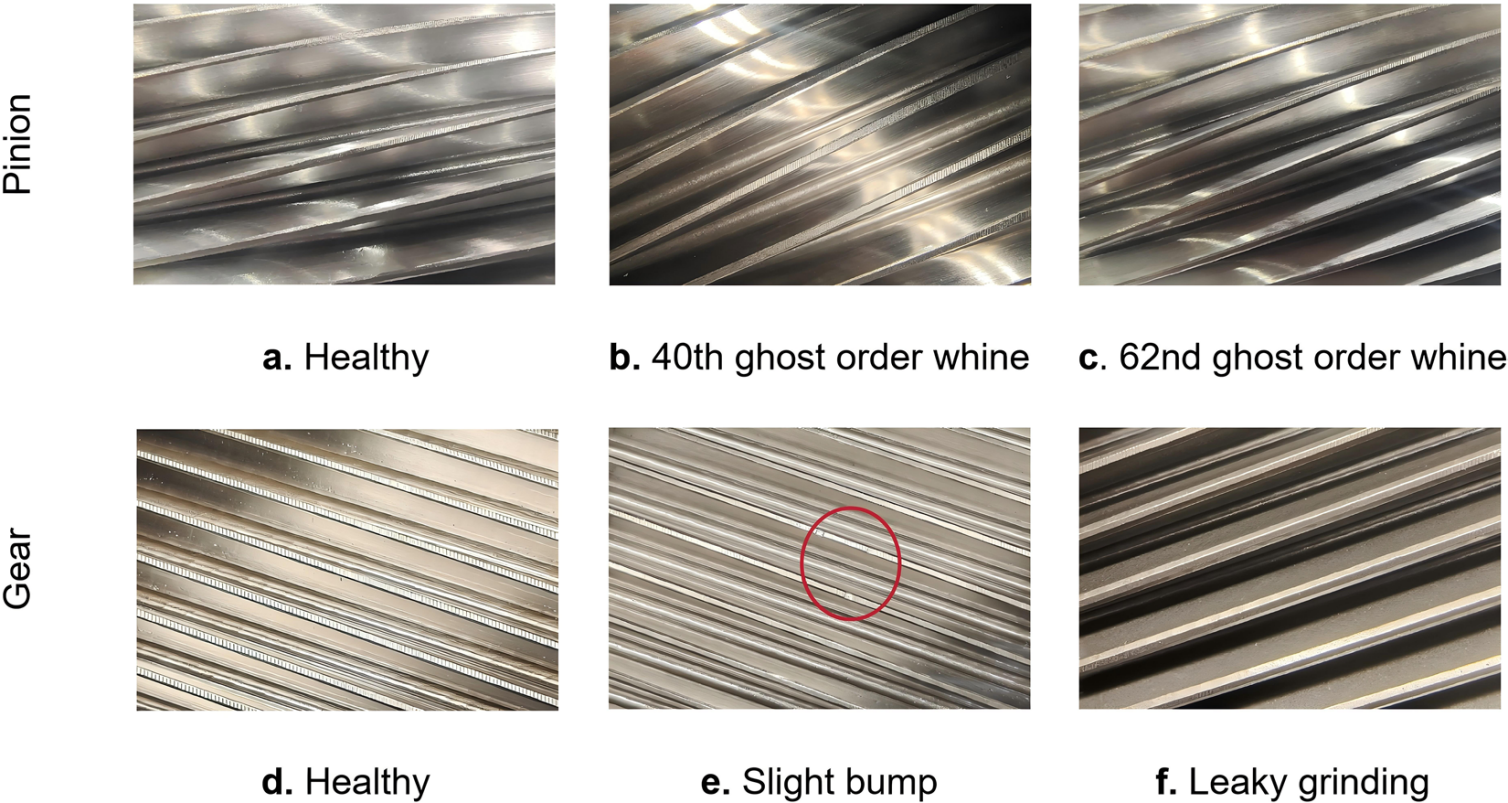
The state of gear before dynamic meshing transmission test.

The DMTD is based on the test data of qualified gears, and includes several unqualified gear states identified in the test. A detailed description of each type of gear is recorded in Table 3. All the test gears are from the manufactured gears to be tested. The state of the gears before testing is shown in Fig. 4. There are small bump marks on the tip of the slight bump gear, and the color of the tooth surface of the leaky grinding gear is slightly darker. There is no obvious observable abnormality in other types of gears.

**Table 3:** A detailed description of the gear type.

	Type	Description	Remarks
0	healthy	—	Both the pinion and gear are qualified.
1	40th ghost order whine	Due to the appearance of regular ripples on the tooth surface after grinding, whine noise occurred during the dynamic meshing transmission test.	The pinion failed, but the gear passed.
2	62nd ghost order whine	Due to the appearance of regular ripples on the tooth surface after grinding, whine noise occurred during the dynamic meshing transmission test.	The pinion failed, but the gear passed.
3	slight bump	Due to the presence of bumps, a regular “tick-tick-tick” noise was emitted within a certain speed range during the meshing transmission test.	The pinion passed, but the gear failed.
4	leaky grinding	After the dynamic meshing transmission test, the gear tooth surface shows a state of ablation.	The pinion is qualified and the gear is not.

**Fig. 5:**
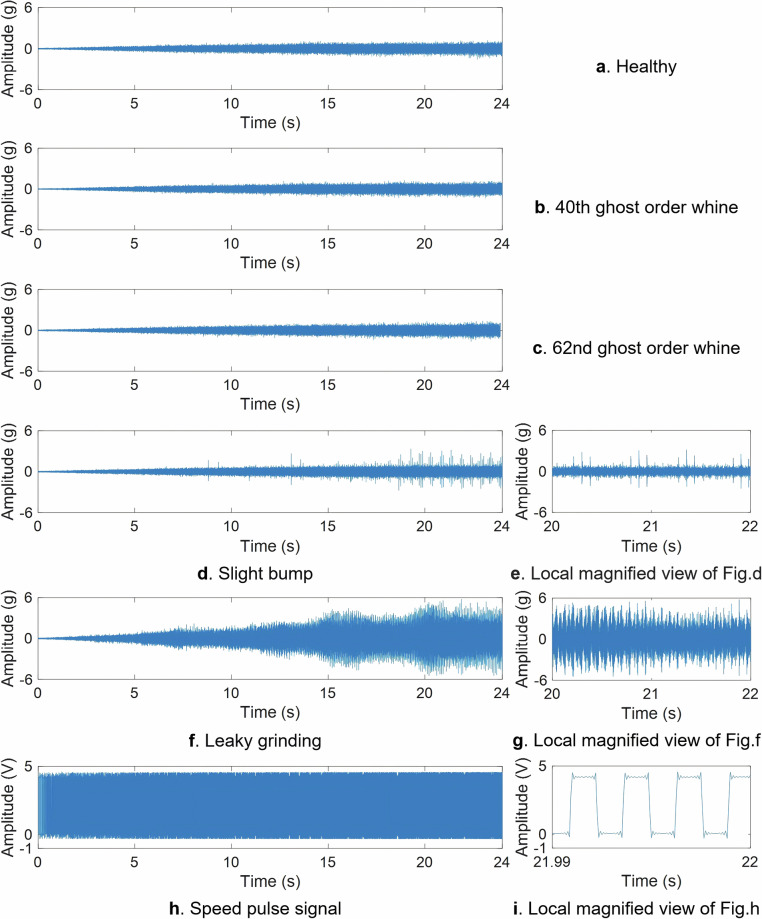
The time domain vibration signals and speed pulse signal under the acceleration condition.

The vibration signals and speed pulse signal in the time domain for each gear type are shown in Fig. 5 (acceleration condition). It can be observed that the time domain signals of slight bump gears and leaky grinding gears have obviously different characteristics from other types. Among them, the slight bump gear has a periodic knock component, and the leaky grinding gear has a long periodic impact component in addition to the short periodic impact component.

## Data Records

**Fig. 6:**
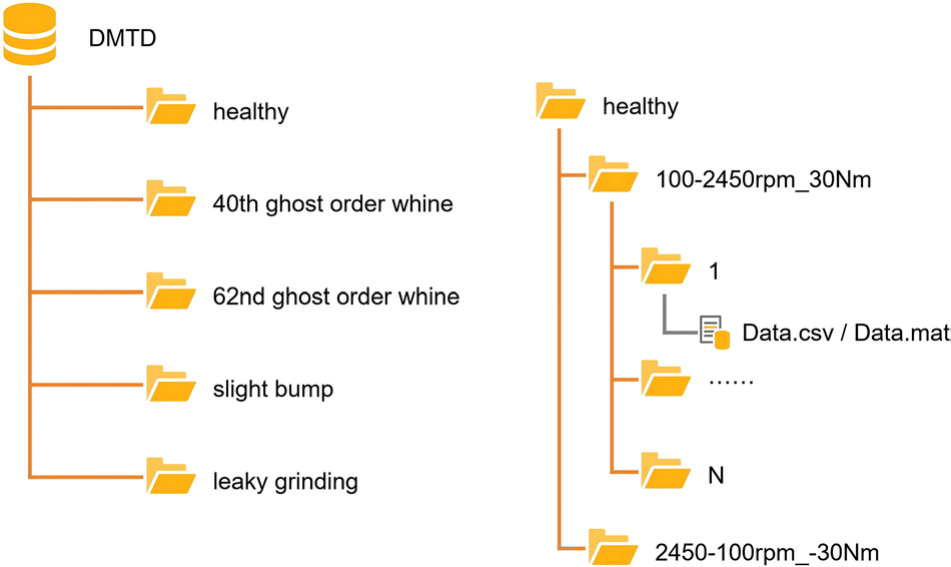
The data directory hierarchy and content of DMTD.

DMTD is available under ScienceDB repositories^3^ in zip compression format. Each gear type contains two folders named after the test condition, and each subfolder of the test condition folder stores a set of vibration and speed pulse data of the test gear. The file format of the original data is “.tdms”. To facilitate public use, we have converted it into “.csv” format and “.mat” format. The naming rule of the data file is “speed range _ load”. For example: “100–2450 rpm_30 Nm” indicates that the data is recorded when the speed range is 100–2,450 r/min and the torque is 30 Nm. The “Data.csv / Data.mat” file contains vibration data and speed pulse data (Each data file contains approximately 614,400 data points: 12,800 (sampling rate) × 24 (about 24 seconds) × 2 (vibration channel and speed pulse channel).). The structure of the dataset is shown in Fig. 6. All test data has been sorted into different folders according to their classification labels and uploaded to the scientific data bank for public access.

## Technical Validation

### Signal processing verification

As described in the “Test gear condition” section, it is difficult to obtain features different from the health type in the time domain for the gear data of the ghost order whine type. Therefore, this work uses the computed order tracking^9^ method to transform the time domain signal into the order domain signal to identify the characteristics of various types of gears more clearly.

**Fig. 7:**
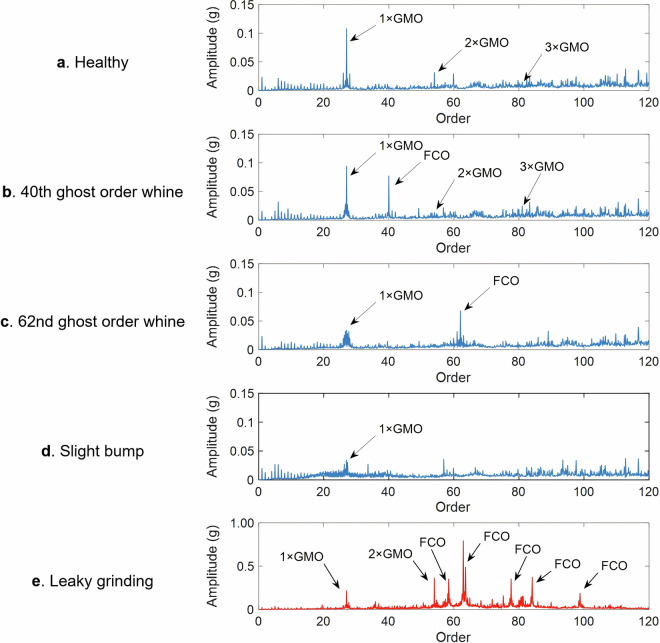
The order domain vibration signals under the acceleration condition.

As shown in Fig. 7, it can be seen that the characteristic distribution of each type of gear in the order domain shows significant differences: the healthy type has clear meshing order characteristics; In addition to the lack of clear meshing order characteristics, the slight bump gear has more complex order component fluctuations; Ghost order whine type gears include the 40th and 62nd ghost order, which are characterized by significant amplitude of ghost order and many harmonic components. The OEM feedback these ghost orders will cause the reducer to produce whine problem; The overall amplitude of the leaky grinding gear is far greater than that of other types (so the red curve is used to draw without maintaining the same scale as other types), and it has an extremely prominent fault feature order. The “GMO” in the figure represents the gear meshing order, and “FCO” represents the fault characteristic order (with the pinion as the reference end, the number of teeth is 27, the meshing order: 1 × GMO = 27, the double meshing order: 2 × GMO = 54).

### Model verification

**Fig. 8:**
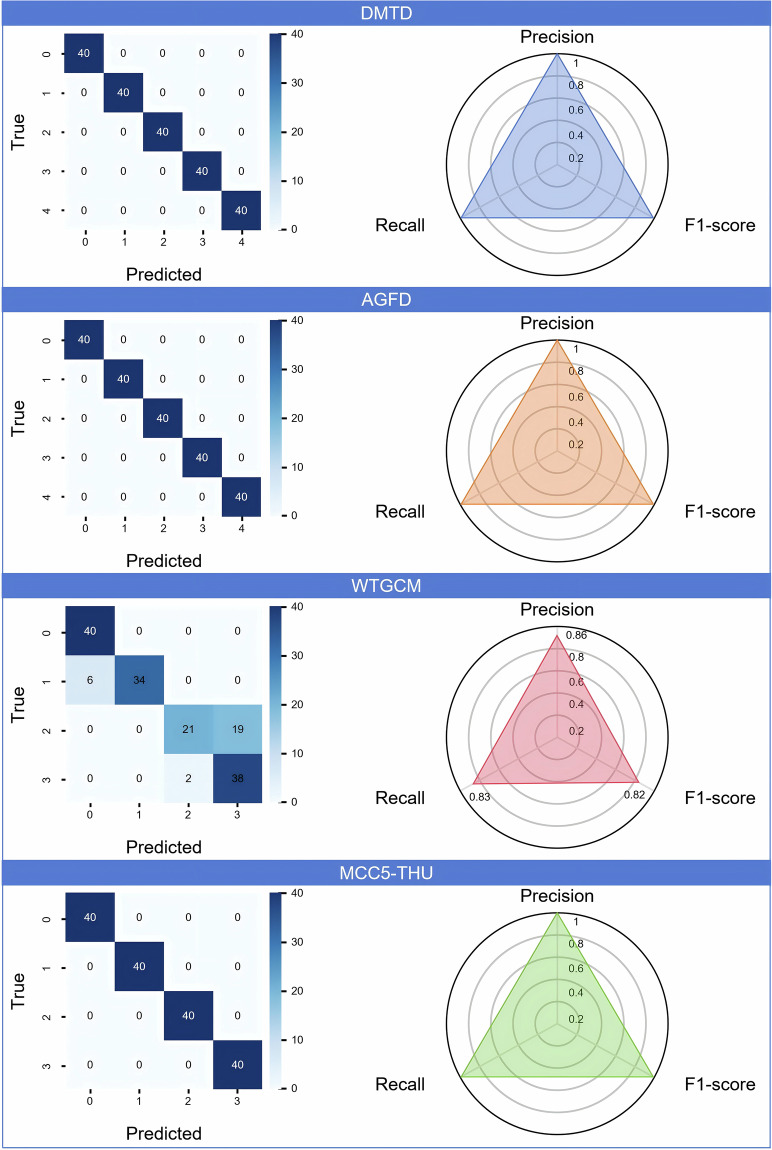
Model verification results.

In order to systematically evaluate the data quality of DMTD, we first intercept the dataset mentioned above into a subsample with a length of 5,000, and then process the order domain dataset by calculating the order tracking method (The data types of each dataset are shown in Table 4). We designed a classifier based on 1D-CNN to train and test all order domain datasets. The experimental results (see Fig. 8) show that compared with the other three datasets, although DMTD has more uncertainty factors, it still maintains competitive performance. This result also verifies the superiority of DMTD in data quality and model generalization ability support.

**Table 4:** Data explanation in model validation.

Dataset	operation conditions	Class 0	Class 1	Class 2	Class 3	Class 4
WTGCM	1,800 r/min	D1_AN7 (Damage)	D1_AN8 (Damage)	H1_AN7 (Healthy)	H1_AN8 (Healthy)	—
MCC5-THU	3,000 r/min 10 Nm	Healthy	Pitting	Wear	Miss teeth	—
AGFD	1,500 r/min 0.71 Nm	Healthy	Mild Micropitting	Mild Pitting	Mild TFF (Tooth Flank Fracture)	Mild Wear
DMTD	100 ~ 2,450 r/min ± 30 Nm	Healthy	40th ghost order whine	62nd ghost order whine	slight bump	leaky grinding

D1 in WTGCM is represented as the first minute data segment of the Damage type data, and AN7 indicates the position of the vibration accelerometer.

## Usage Notes

To read and analyze the DMTD, it is recommended to use MATLAB or Python for data processing. Additionally, in order to correctly convert the speed pulse signal into revolutions per minute (r/min), the Pulses Per Revolution (PPR) should be set to 10. At the same time, it is suggested to use a 3.5-volt threshold to detect the rise and fall of the signal. When performing Fourier transformation, it is recommended to use the Hanning window.

## Data Availability

DMTD can be accessed unrestrictedly in the ScienceDB repository via the following link: 10.57760/sciencedb.27983.

## Code availability

The model code used in this work can be accessed through the following link:

10.57760/sciencedb.27983.
